# Protocol to use TopNet for gene regulatory network modeling using gene expression data from perturbation experiments

**DOI:** 10.1016/j.xpro.2022.101737

**Published:** 2022-09-30

**Authors:** Helene R. McMurray, Harry A. Stern, Aslihan Ambeskovic, Hartmut Land, Matthew N. McCall

**Affiliations:** 1 Department of Biomedical Genetics, University of Rochester Medical Center, 601 Elmwood Avenue, Rochester, NY 14642, USA; 2 Department of Pathology and Laboratory Medicine, University of Rochester Medical Center, 601 Elmwood Avenue, Rochester, NY 14642, USA; 3 Center for Integrated Research Computing, University of Rochester Medical Center, 601 Elmwood Avenue, Rochester, NY 14642, USA; 4 Wilmot Cancer Institute, University of Rochester Medical Center, 601 Elmwood Avenue, Rochester, NY 14642, USA; 5 Department of Biostatistics and Computational Biology, University of Rochester Medical Center, 601 Elmwood Avenue, Rochester, NY 14642, USA

**Keywords:** Bioinformatics, Cell Biology, Cell culture, Cancer, Genetics, Molecular Biology, Gene Expression, Systems biology

## Abstract

Inference of gene regulatory networks from gene perturbation experiments is the most reliable approach for investigating interdependence between genes. Here, we describe the initial gene perturbations, expression measurements, and preparation steps, followed by network modeling using TopNet. Summarization and visualization of the estimated networks and optional genetic testing of dependencies revealed by the network model are demonstrated. While developed for gene perturbation experiments, TopNet models data in which nodes are both perturbed and measured.

For complete details on the use and execution of this protocol, please refer to McMurray et al. (2021).

## Highlights

• This protocol describes the use of the TopNet algorithm to model gene networks using perturbation data
• Techniques to summarize and visualize the resulting gene networks are described and demonstrated
• Necessary experimental and bioinformatic steps to generate the input data for the TopNet algorithm are described
• Examples for genetic testing of dependencies uncovered by the network modeling as well as troubleshooting of both experimental and computational techniques are provided

## Abstract (editor highlights)

Publisher’s note: Undertaking any experimental protocol requires adherence to local institutional guidelines for laboratory safety and ethics.

## Graphical abstract

**Figure:**
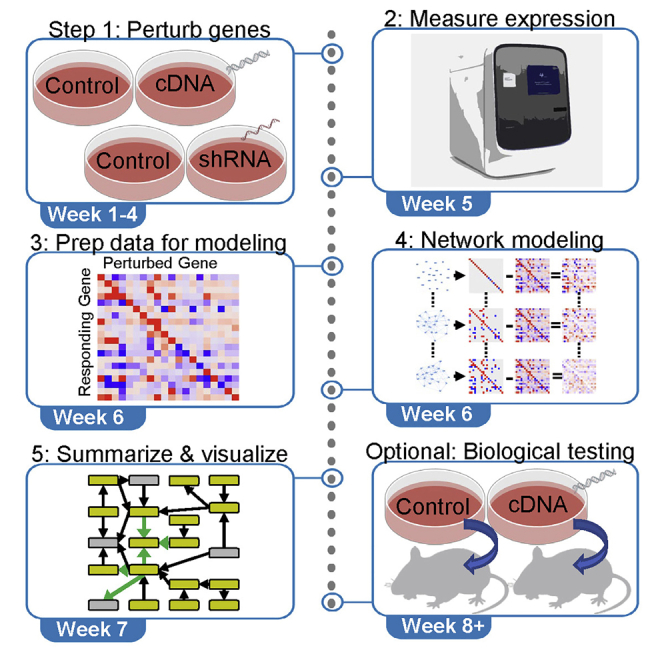

## Before you begin

We have divided this section into two parts. First, we describe the cell culture. Next, we describe the computational setup.

### Cell culture

The protocol below describes the specific steps for using young adult mouse colon (YAMC) cells derived from the Immorto-mouse (also known as the H-2Kb/tsA58 transgenic mouse) (Jat et al., 1991; Whitehead et al., 1993). These cells express temperature-sensitive simian virus 40 large T (tsA58 mutant) under the control of an interferon γ-inducible promoter and have been used by our group to enable study of oncogene cooperation in colon cells. However, we have also adapted this protocol for use in human colorectal cancer cells lines DLD-1, HT-29, SW480, SW620, human prostate cancer cell line PC3 and human breast cell lines MCF10A and MDA-MB-231 cells, using appropriate culture conditions for each of those cell lines.

### Institutional permissions

A material transfer agreement (MTA) is required to obtain young adult mouse colon cells. We obtained these cells under an MTA from R. Whitehead and A.W. Burgess, who originally derived the cell populations from the Immorto-mouse as described in their published manuscripts (Jat et al., 1991; Whitehead et al., 1993).

### Culture cells to be perturbed, exemplified here for young adult mouse colon (YAMC) cells

**Timing: 2 h for routine passage of cells, multiple times per week for ongoing cell culture**

YAMC cells expressing dominant negative mutant p53 (p53^175H^) and constitutively active mutant Ras (Ras^V12^) were derived by retroviral infection of low-passage polyclonal YAMC cells in the Land laboratory (McMurray et al., 2008; Xia and Land, 2007). These cells are referred to as mp53/Ras cells in this and related publications. Here we describe the generation of these cells.

YAMC and mp53/Ras cells are maintained at the permissive temperature (33°C) in the presence of interferon γ to allow expression and function of the temperature-sensitive SV40 large T transgene in vitro. This permits expansion of the parental YAMC cells in tissue culture. When YAMC cells are switched to the non-permissive temperature for large T (39°C) in the absence of interferon γ, their growth rapidly arrests followed by cell death, indicating the absence of spontaneous immortalizing mutations in the cell population. In contrast, the expression of both p53^175H^ and Ras^V12^ together in the mp53/Ras cells drives cancerous transformation, allowing them to grow long-term at the non-permissive temperature (39°C).

### Coat dishes with collagen to support YAMC cell growth

**Timing: 2 h for routine passage of cells, multiple times per week for ongoing cell culture**

Parental YAMC cells require extracellular matrix support for optimal growth. Thus, we routinely culture these cells on collagen-coated tissue culture (TC) dishes. Dishes are coated at 1 μg/cm^2^. While not required for growth of the mp53/Ras cells, both YAMC and mp53/Ras cells were grown on collagen-coated dishes for consistency of handling.

1. Prepare collagen stock solution at 50× in 0.25% sterile acetic acid. Prepared solution can be stored at 4°C for up to one month.
2. Dilute collagen stock solution to 1× with sterile PBS. Add 3 mL diluted collagen to each 10-cm dish or 6 mL to 15-cm dishes. Rock the dishes to distribute the liquid across the entire surface of each dish. Incubate at room temperature (20°C–24°C) for 90 min.
3. At the end of this incubation, aspirate excess collagen solution. Add sterile 1× PBS. to each plate, using 5 mL per 10 cm dish or 10 mL per 15 cm dish.
  a. Plates can be used immediately or stored at 33°C or 37°C for up to 5 days. Plates should be stored containing 10 mL sterile 1× PBS (i.e., don’t aspirate the PBS wash solution until ready to use the plate).

***Note:*** We have used rat tail collagen, type I (Becton Dickinson, #354236) and human placental collagen, type IV (Sigma) with similar results in terms of cell growth.

***Note:*** We obtain the best results with Corning TC dishes.

### Prepare media for routine cell culture at 33°C

**Timing: ∼30 min each time new bottles of media need to be prepared**

4. Prepare no more media than necessary as the pH will change over time due to exposure to room air. We generally prepare 500 mL at a time (one bottle as purchased).
5. For 33°C culture, the following should be added to RPMI 1640 medium:
  a. ∼9% fetal bovine serum (FBS).
  b. 1× ITS-A.
  c. 2.5 μg/mL gentamycin.
  d. 5 U/mL interferon γ.
  e. Prepared media can be stored at 4°C for up to one month.

***Note:*** Interferon γ stocks are made at 250 U/μL in 1× PBS containing 0.1% BSA. The resulting solution is filter sterilized and stored at −20°C for up to 6 months.

***Optional:*** Thaw frozen aliquots of YAMC cells or derivatives for routine culture at 33°C

6. Remove cell aliquots from liquid nitrogen storage.
  a. **Loosen tube caps to prevent explosion from escaping nitrogen**, but do not remove caps completely as this would compromise cell sterility.
    i. YAMC cells and derivatives are frozen in 33°C media supplemented with 10% DMSO.
    ii. Cells are never frozen from 39°C culture conditions.
    iii. Aim to freeze 10^6^ – 5 × 10^6^ cells in 1 mL of freezing media per vial, allowing these to be thawed onto 10 cm TC dishes.
7. Float tubes in 37°C water bath for 2 min. Remove tubes from water and pat dry.
8. Remove entire volume of cells and media and add to 10 mL media on a collagen-coated dish.
9. Place plated cells into 33°C tissue culture incubator with 5% CO2. After cells are allowed to adhere to dishes, between 16 – 24 h after plating, aspirate media and replace with fresh 33°C media to remove residual DMSO from thawed cells.

***Note:*** YAMC cells and derivatives are frozen in 33°C media supplemented with 10% DMSO. Cells are never frozen from 39°C culture conditions. Aim to freeze 10^6^ – 5 × 10^6^ cells per vial, allowing these to be thawed onto 10 cm TC dishes.

### Perform routine maintenance of cultured cells

10. Every 3–5 days, YAMC parental cells are split at a ratio of 1:2 to 1:4 and mp53/Ras cells are split at a ratio of 1:8 to 1:12.
  a. Determination of when to split is done by visual inspection of cell density on plates. YAMC cells do best when kept between 40 – 90% confluent.
    i. Plating too sparsely may cause YAMC cells to die.
    ii. Plating too densely may cause YAMC cells to undergo growth arrest due to contact inhibition.
  b. By virtue of having undergone malignant transformation, mp53/Ras cells are not as sensitive to cell density on dishes. Moreover, they divide faster than YAMC cells and thus are better handled by splitting at higher ratios.
11. To split cells, trypsinize using 1× trypsin-EDTA (Invitrogen, #25300) at room temperature for 5–10 min. Dilute with 33°C culture media to inhibit trypsin, then plate on unused, collagen coated TC dishes at ratios described in step 10.

***Note:*** Cells can also be counted and plated at defined densities as when temperature switching by plating the cells in media for growth at 39°C (described in step-by-step method details, steps 12 and 13).

### Collect necessary perturbagens, exemplified here by production of retroviral vectors harboring cDNA or shRNA molecules

**Timing: ∼1 week**

12. Thaw and culture ΦΝΧ-E viral packaging cells (also known as Phoenix-ECO cells, available from ATCC) in Dulbecco’s Modified Eagle Medium (DMEM) supplemented with 10% FBS, 50 μg/mL kanamycin and 2.5 μg/mL gentamicin.
  a. These cells are derivatives of HEK293T and contain stably integrated plasmids to allow mouse-infectious retroviral vector production.
  b. Moreover, they are amenable to transfection with lipid-based transfection reagents or with calcium phosphate. The latter is our preferred protocol, outlined below.
13. Prepare DNA for each perturbation that will be performed in a given experiment.
  a. A simple experiment would include two plasmids, each used to transfect and infect an independent plate of cells. For example, you would use a pBabe construct containing cDNA for a gene of interest and an empty vector control (i.e., pBabe with no insert).
  b. Plasmid DNA encoding retroviral constructs, such as pBabe vectors encoding selected cDNA or pSuper-retro vectors expressing selected shRNA, should be obtained by carrying out the manufacturer’s protocol (QIAGEN) for maxi-prep of recombinant *E. coli* carrying the appropriate plasmids.
  c. Once prepped, plasmid DNA can be stored at −20°C and utilized for multiple experiments for up to one year.
14. To generate virus for infection of YAMC or mp53/Ras cells, plate ΦΝΧ-E on a 10 cm dish at a density of 2.5 × 10^6^ cells in 5 mL of media, at least 6 and no more than 24 h prior to transfection.
  a. Set up individual plates for each population of virus to be generated (i.e., make separate plates for vector controls and for each perturbation vector that will be generated).
15. Prepare fresh stock solutions of 2 M CaCl_2_ and 2× HBS (recipe below). Filter sterilize both solutions. Do not store or re-use excess.
  a. 2× HBS contains 250 mM NaCl, 50 mM HEPES, pH 8.0 and 1.5 mM Na_2_HPO_4_.
  b. Adjust pH to between 7.1 to 7.3 by adding 1 N NaOH dropwise until desired pH is achieved.

**CRITICAL:** Correct pH of this solution is key to transfection efficiency.

16. For 10 cm dishes, mix 20 μg DNA, 25 μL of 2 M CaCl_2_ and H_2_O to a final volume of 200 μL.
  a. This makes the final CaCl_2_ concentration 0.25 M.
17. Dilute 2× HBS to 1× (1:1 v/v) with the DNA/CaCl_2_/H_2_O mix from step 17.
  a. CRUCIAL: Add DNA solution to the HBS by blowing air bubbles into mix or vortexing gently as you add DNA solution dropwise.
  b. For example, with 200 μL DNA mix, add 200 μL 2× HBS.
18. Incubate at room temperature for 15 min.
  a. The solution should become cloudy as precipitate forms in the tubes.
19. Add CaCl_2_/DNA/HBS mix dropwise to ΦΝΧ-E plates.
20. Incubate at 37°C overnight (12–16 h). Change media, replacing with fresh media (as in step 12) in 5 mL volume per 10 cm dish.
21. At 40 and 43–46 h post-transfection (∼1 day after changing the media), collect the supernatant that will contain viral particles.
  a. Virus can be used immediately for infection of YAMC or mp53/Ras cells, our preferred method.
    i. If using fresh virus, you MUST filter the virus through 0.45 μm cellulose acetate filters (Pall) to remove potentially contaminating ΦΝΧ-E cells.
  b. Virus can also be aliquoted and frozen at −80°C to store. No special measures are necessary in this case, as the process of freezing will kill any ΦΝΧ-E cells that may be in the supernatants.

### Computational setup

We now describe the steps necessary to install the software packages used by the TopNet algorithm.

### Install the ternarynet R/Bioconductor package used by TopNet for network modeling

**Timing: <1 h**

The TopNet network modeling algorithm is distributed as part of the **ternarynet** package in Bioconductor (see https://bioconductor.org/packages/release/bioc/html/ternarynet.html). The **ternarynet** package contains a serial algorithm, that uses simulated annealing, and a parallel algorithm, that uses replica exchange Monte Carlo. Either of these algorithms can be used by the TopNet algorithm for network modeling.

22. To install the serial implementation of **ternarynet**, which uses simulated annealing, install R version 4.1 or later and enter:

if (!require("BiocManager", quietly = TRUE))

 install.packages("BiocManager")

BiocManager::install("ternarynet")

***Optional:*** The parallel (replica exchange) implementation of ternarynet requires openMPI (version 1.x, from https://www.open-mpi.org), the R packages Rmpi and snow (available from https://cran.r-project.org/), and BiocParallel (from BioConductor). First, verify that you have autoconf installed or install it from https://www.gnu.org/software/autoconf/. Second, the ternarynet package source code should be downloaded and unpacked. Then the following commands should be given at the command line, where 1.x is replaced with the actual version of openMPI used:

autoconf

R CMD build ternarynet

R CMD INSTALL --clean ternarynet_1.38.0.tar.gz --configure-args='--with-mpi=/path/to/mpi-1.x --with-Rmpi-type=OPENMPI'

This will allow the **parallelFit** function in **ternarynet** to use replica exchange Monte Carlo for network estimation. Since **parallelFit** uses MPI, R must be launched at the command line using mpirun. For example:

mpirun -n 1 R --vanilla < network_fit.R

where the script network_fit.R contains a call to parallel fit.

## Key resources table

**Table:** 

REAGENT or RESOURCE	SOURCE	IDENTIFIER
**Bacterial and virus strains**		

pBabe retroviral vector containing either puromycin or hygromycin resistance genes	Addgene	pBabe-puro (#1764) pBabe-hygro (#1765)
pSuper retroviral vector containing puromycin resistance gene	Oligoengine	pSuper.retro.puro (VEC-pRT-0002)
pLKO lentiviral vector containing puromycin resistance gene	Addgene	pLKO.1 puro (#8453)
pLenti/UbC/V5 lentiviral vector containing puromycin resistance gene	Invitrogen	pLenti6/UbC/V5 (V49910)

**Chemicals, peptides, and recombinant proteins**		

RPMI 1640 media	Invitrogen	11875119
Fetal bovine serum	HyClone	SH30071
ITS-A	Invitrogen	51300044
Gentamycin	Invitrogen	15750060
Interferon gamma	R&D Systems	485MI
Rat tail derived collagen IV	Corning	354236
Polybrene	Sigma-Aldrich	H9268
Puromycin	Sigma-Aldrich	P7255
Hygromycin	Invitrogen	10687010
Blasticidin	Invitrogen	R21001

**Critical commercial assays**		

RNeasy Mini Kit with on-column DNase digestion	QIAGEN	74106
High Capacity cDNA Reverse Transcription Kit	Applied Biosystems	4368814
iQ SYBR Green qPCR Master Mix	Bio-Rad	1708884
TaqMan probe detecting murine *Death-associated protein kinase 1 (Dapk1)*	Applied Biosystems	Mm00459400_m1
TaqMan probe detecting murine *DNA fragmentation factor, beta subunit (Dffb)*	Applied Biosystems	Mm00432822_m1
TaqMan probe detecting murine *Fas (TNF receptor superfamily member 6) (Fas)*	Applied Biosystems	Mm00433237_m1
TaqMan probe detecting murine *Homeobox C13 (HoxC13)*	Applied Biosystems	Mm00802798_m1
TaqMan probe detecting murine *Inhibitor of DNA binding 2 (Id2)*	Applied Biosystems	Mm00711781_m1
TaqMan probe detecting murine *Inhibitor of DNA binding 4 (Id4)*	Applied Biosystems	Mm00499701_m1
TaqMan probe detecting murine *Jagged 2 (Jag2)*	Applied Biosystems	Mm00439935_m1
TaqMan probe detecting murine *Notch 3 (Notch3)*	Applied Biosystems	Mm00435270_m1
TaqMan probe detecting murine *Phorbol-12-myristate-13-acetate-induced protein 1 (Noxa, also known as Pmaip1)*	Applied Biosystems	Mm00451763_m1
TaqMan probe detecting murine *Par-6 family cell polarity regulator gamma (Pard6g)*	Applied Biosystems	Mm00474139_m1
TaqMan probe detecting murine *PERP, TP53 apoptosis effector (Perp)*	Applied Biosystems	Mm00480750_m1
TaqMan probe detecting murine *Phospholipase A2, group VII (Pla2g7)*	Applied Biosystems	Mm00479105_m1
TaqMan probe detecting murine *Placenta-specific 8 (Plac8)*	Applied Biosystems	Mm00507371_m1
TaqMan probe detecting murine *Rab40B, member RAS oncogene family (Rab40b)*	Applied Biosystems	Mm00454800_m1
TaqMan probe detecting murine *Regulator of G-protein signaling 2 (Rgs2)*	Applied Biosystems	Mm00501385_m1
TaqMan probe detecting murine *Reprimo, TP53 dependent G2 arrest mediator candidate (Rprm)*	Applied Biosystems	Mm00469773_s1
TaqMan probe detecting murine *Secreted frizzled-related protein 2 (Sfrp2)*	Applied Biosystems	Mm00485986_m1
TaqMan probe detecting murine *Spermine synthase (Sms)*	Applied Biosystems	Mm00786246_s1
TaqMan probe detecting murine *Wingless-type MMTV integration site family, member 9A (Wnt9a)*	Applied Biosystems	Mm00460518_m1
TaqMan probe detecting murine *Zinc finger protein 385A (Zfp385)*	Applied Biosystems	Mm00600201_m1

**Experimental models: Cell lines**		

Young adult mouse colon cells derived from the H-2Kb / tsA58 transgenic mouse	Gift of R. Whitehead and A.W. Burgess	YAMC cells
“Phoenix” cells producing murine ecotropic virus (ΦΝΧ-ECO or Phoenix-ECO)	ATCC	Phoenix-ECO
Human embryonic kidney 293 cells expressing SV40 T antigen	ATCC	HEK 293T

**Experimental models: Organisms/strains**		

Crl: CD-1-Foxn1^nu^ female mice	Charles River Laboratories	Crl: CD-1-Foxn1^nu^

**Oligonucleotides**		

Dapk1 Forward Primer	IDT	GGAGACACCAAGCAAGAAA
Dapk1 Reverse Primer	IDT	ACAAGGAGCCCAGGAGAT
Dffb Forward Primer	IDT	ACCCAAATGCGTCAAGTT
Dffb Reverse Primer	IDT	GCTGCTTCATCCACCATA
Fas Forward Primer	IDT	CCGAGAGTTTAAAGCTGAGG
Fas Reverse Primer	IDT	CCAGGAGAATCGCAGTAGAAGTCTGG
HoxC13 Forward Primer	IDT	GCTAAGGAGTTCGCCTTCTACC
HoxC13 Reverse Primer	IDT	CCAGCCATTGGAAAGAGCC
Id2 Forward Primer	IDT	CGGTGAGGTCCGTTAGGAAAA
Id2 Reverse Primer	IDT	CATGTTGTAGAGCAGACTCATCG
Id4 Forward Primer	IDT	CAGTGCGATATGAACGACTGC
Id4 Reverse Primer	IDT	GACTTTCTTGTTGGGCGGGAT
Jag2 Forward Primer	IDT	TGTGACGAGTGTGTCCCCTA
Jag2 Reverse Primer	IDT	GCGCAGAGGTATTGGTCAGG
Notch3 Forward Primer	IDT	CTGCCAAAGTGACATAGATGAGT
Notch3 Reverse Primer	IDT	GCCCTGTATAACCAAGAGGACA
Noxa (Pmaip1) Forward Primer	IDT	TGAGTTCGCAGCTCAACTC
Noxa (Pmaip1) Reverse Primer	IDT	TCAGGTTACTAAATTGAAGAGCTTGGAAATC
Pard6g Forward Primer	IDT	AGTGCAAACCCCTTGCTTC
Pard6g Reverse Primer	IDT	GCAGACCGTCATCCCTCAG
Perp Forward Primer	IDT	ATGGAGTACGCATGGGGAC
Perp Reverse Primer	IDT	GATTACCAGGGAGATGATCTGGA
Pla2g7 Forward Primer	IDT	ATCAAGGTCGCCTCGACAC
Pla2g7 Reverse Primer	IDT	GCAGGAGTTGTCAGAGAACCAT
Plac8 Forward Primer	IDT	GCTCAGGCACCAACAGTTATC
Plac8 Reverse Primer	IDT	GTTCCACACAGACAACACTC
Rab40b Forward Primer	IDT	GTGCGGGCCTACGATTTTCTA
Rab40b Reverse Primer	IDT	GTGGCCGTAAGGAGACTCG
Rgs2 Forward Primer	IDT	ACCAAATCACCCCAAAAACTGT
Rgs2 Reverse Primer	IDT	GCCACTTGTAGCCTCTTGGAT
Rprm Forward Primer	IDT	GTGTGGTGCAGATCGCAGT
Rprm Reverse Primer	IDT	ATCATGCCTTCGGACTTGATG
Sfrp2 Forward Primer	IDT	GGCCACGAGACCATGAAGG
Sfrp2 Reverse Primer	IDT	GAAGAGCGAGCACAGGAACT
Sms Forward Primer	IDT	CAGCTTTGCCAATTTGCGAAT
Sms Reverse Primer	IDT	CTATGGGTGGTAATCGCTTCAC
Wnt9a Forward Primer	IDT	GGTGGGCAAGCACCTAAAAC
Wnt9a Reverse Primer	IDT	GTACAAGCTCTGGTGTTCGGG
Zfp385 Forward Primer	IDT	CTACAAGGGTAATCGCCATGC
Zfp385 Reverse Primer	IDT	GTCCCGACTCTGGAACACTG

**Recombinant DNA**		

pBabe-puro-Dapk1	Constructed for this study	
pBabe-puro-Dffb	Constructed for this study	
pBabe-puro-Fas	Constructed for this study	
pBabe-puro-HoxC13	Constructed for this study	
pBabe-hygro-HoxC13	Constructed for this study	
pBabe-puro-Id2	Constructed for this study	
pBabe-puro-Id4	Constructed for this study	
pBabe-puro-Jag2	Constructed for this study	
pBabe-puro-Notch3	Constructed for this study	
pBabe-puro-Noxa	Constructed for this study	
pBabe-puro-Pard6g	Constructed for this study	
pBabe-puro-Perp	Constructed for this study	
pSuper.retro.puro-Pla2g7	Constructed for this study with target sequence: GGCCGTCAGTAATGTTTCA	
pSuper.retro.puro-Plac8	Constructed for this study with target sequence: GTGGCAGCTGACATGAATGTT	
pBabe-puro-Plac8	Constructed for this study	
pBabe-puro-Rab40b	Constructed for this study	
pSuper.retro.puro-Rgs2	Constructed for this study with target sequence: GGCTGTGACCTGCCAGAAA	
pBabe-puro-Rgs2	Constructed for this study	
pBabe-puro-Rprm	Constructed for this study	
pBabe-puro-Sfrp2	Constructed for this study	
pSuper.retro.puro-Sfrp2	Constructed for this study with target sequence: CCTAACATGTCCTGAG	
pSuper.retro.puro-Sfrp2	Constructed for this study with target sequence: TGGTCAGTCTGTTGGC	
pSuper.retro.puro-Sms	Constructed for this study with target sequence: CGATCCACAACCTATTATA	
pSuper.retro.puro-Sms	Constructed for this study with target sequence: AGACAGCCCAGCAAAGACT	
pLenti6/UbC/V5-Sms	Constructed for this study	
pBabe-puro-Wnt9a	Constructed for this study	
pBabe-hygro-Wnt9a	Constructed for this study	
pBabe-puro-Zfp385	Constructed for this study	

**Software and algorithms**		

Sequence Detection System software	Applied Biosystems	SDS v2.0, SDS v3.0
Cytoscape	Cytoscape Consortium	https://cytoscape.org/
Non-detect imputation algorithm (nondetects package)	Bioconductor	https://doi.org/10.18129/B9.bioc.nondetects
TopNet algorithm (ternarynet package)	Bioconductor	https://doi.org/10.18129/B9.bioc.ternarynet
Reproducible workflow (crgnet package)	This paper	https://doi.org/10.5281/zenodo.7047161
Pseudocode for the network modeling algorithm	This paper	Methods S1

## Materials and equipment

**Table:** Media for growth of YAMC or mp53/Ras Cells at 33°C

Reagent	Final concentration	Amount
RPMI 1640 medium (Invitrogen)	N/A	500 mL
Fetal bovine serum (FBS, Hyclone)	9%	50 mL
Insulin / Transferrin / Selenium / Sodium Pyruvate (ITS-A, Invitrogen)	0.9×	5 mL
Gentamycin (Invitrogen, 25 mg/mL stock)	2.25 μg/mL	50 μL
Interferon gamma (IFN-γ, R&D Systems, 250 U/μL stock)	4.5 U/mL	10 μL
**Total**		**555.06 mL**

Prepared media should be stored at 4°C. Media can be used for up to one month if no signs of bacterial growth are seen and the pH is in the range from approximately 7.2 to 7.6.

***Alternatives:*** If cells other than YAMC parental or derived lines are being grown, use appropriate culture media for the selected cells.

**Table:** Media for growth of YAMC or mp53/Ras Cells at 39°C

Reagent	Final concentration	Amount
RPMI 1640 medium (Invitrogen)	N/A	500 mL
Fetal bovine serum (FBS, Hyclone)	∼10%	50 mL
Insulin / Transferrin / Selenium / Sodium Pyruvate (ITS-A, Invitrogen)	1×	5 mL
Gentamycin (Invitrogen, 25 mg/mL stock)	2.5 μg/mL	50 μL
**Total**		**555.05 mL**

Prepared media should be stored at 4°C. Media can be used for up to one month if no signs of bacterial growth, such as turbidity or odor, are noted and the pH is in the range from approximately 7.2 to 7.6.

***Alternatives:*** As above for 33°C media.

**Table:** Media for serum starvation of YAMC or mp53/Ras Cells at 39°C

Reagent	Final concentration	Amount
RPMI 1640 medium (Invitrogen)	N/A	500 mL
Insulin / Transferrin / Selenium / Sodium Pyruvate (ITS-A, Invitrogen)	1×	5 mL
Gentamycin (Invitrogen, 25 mg/mL stock)	2.5 μg/mL	50 μL
**Total**		**505.05 mL**

Prepared media should be stored at 4°C. Media can be used for up to one month if no signs of bacterial growth are seen and the pH is in the range from approximately 7.2 to 7.6.

***Alternatives:*** As above for 33°C media.

**Table:** 2× HBS for Use in Calcium Phosphate-Mediated Transfection

Reagent	Final concentration	Amount
Sodium Chloride (NaCl, 2.5 M stock)	250 mM	2 mL
HEPES, pH 8.0 (1 M stock)	50 mM	1 mL
Disodium Hydrophosphate (Na_2_HPO_4,_ 200 mM stock)	1.5 mM	150 μL
**Total**	**N/A**	**20 mL**

This solution must be prepared fresh each time transfections will be performed.

**CRITICAL:** Adjust pH to 7.1–7.3. The pH of this solution is the key to transfection efficiency. Filter sterilize.

***Alternatives:*** Lipid-mediated transfection methods may be used as an alternative to calcium phosphate transfection.

## Step-by-step method details

### Perturb gene expression in mp53/Ras cell populations by stable retroviral transduction and drug selection

**Timing: Two to three weeks**

This step creates cell populations with expression of desired cDNA or knock-down of desired target gene by shRNA. The derived cells are used for additional experiments described below.

1. One day prior to infection, plate mp53/Ras cells at 33°C.
  a. Use one plate for each perturbation to be derived, including an empty vector control each time infections are performed.
  b. Make an additional plate of cells for “mock” infection (no virus added).
    i. The mock infection allows monitoring of drug selection. Populations infected with retrovirus should have surviving cells, while the “mock” infected cells should all die in the presence of drug.
  c. Plate mp53/Ras cells at 250,000 cells / 10 cm collagen-coated dish.
2. Prepare polybrene (also known as hexadimethrine bromide) in PBS at desired stock concentration.
  a. For 100×, dissolve 800 μg/mL. For 1000×, dissolve 8 mg/mL.
  b. Polybrene can be stored at 4°C for up to 6 months or can be frozen at –20°C for longer periods.
3. One day after plating cells (18–24 h), aspirate media from plates and pipet collected supernatants containing viruses on to each plate to be infected.
  a. Retrovirus-containing supernatants should have a 5 mL volume if generated as described in “before you begin” steps 20 and 21 above.
  b. Use only one type of virus per plate of cells.
4. For mock infection, replace media with 5 mL of fresh 33°C media.
5. Add 8 μg of polybrene per mL of supernatant to each dish being infected (i.e., dilute 100× or 1000× stock to 1× final concentration in volume of supernatant on each dish).
6. Incubate for 1.5–3 h at 33°C in CO_2_ incubator.
7. Aspirate supernatant and replace with second aliquot of supernatant for virus containing the same perturbagen. In other words, the same dish should receive multiple rounds of infection with empty vector or virus harboring a single cDNA or shRNA insert.
  a. Generally, we do 2 rounds of infection but YAMC and mp53/Ras cells can tolerate up to 6 rounds of infection before experiencing toxic effects from exposure to polybrene.
8. Following all desired rounds of infection, replace supernatant with fresh 33°C media.
9. Allow cells to recover for 48–72 h prior to beginning drug selection.
10. For drug selection of YAMC or mp53/Ras cells, i.e., elimination of uninfected cells in the population, add the appropriate selective agent to 33°C media at concentrations listed below and depending on the resistance marker present in the retroviral vector used.
  a. Puromycin: 5 μg/mL media; Selection takes 2–4 days.
  b. Hygromycin: 200 μg /mL media; Selection takes 4–7 days.
  c. Bleomycin: 100–400 μg /mL media; Selection takes up to 2 weeks.
  d. Neomycin/Geneticin: 100–400 μg /mL media; Selection takes up to 2 weeks.
11. Once perturbed populations are derived, they should be maintained in media with the appropriate selective agent during routine maintenance / cell splitting.

**CRITICAL:** Selected cell populations should be used for RNA isolation, tumor formation studies and other experimentation within two weeks of derivation. Long-term culture of perturbed populations can allow for drift in the population and altered cell behavior over time in culture.

***Note:*** For experiments described in McMurray et al., Cell Reports, 2021, we freshly transduced and selected polyclonal populations for each biological replicate and each perturbation described in the paper.

***Note:*** For double / combined perturbation, derive polyclonal population harboring one perturbation and then repeat above steps for second perturbagen. Be sure to use perturbagens with distinct selectable markers to enable selection of pure populations of perturbed cells harboring both perturbations. When selecting with multiple selective agents, use half the dosage recommended above to avoid non-specific cell killing.

### Extract RNA and measure gene expression in perturbed mp53/Ras cell populations

**Timing: 5–7 days**

This step involves re-plating perturbed cell populations for short-term growth at 39°C and then starving them of serum to remove the influence of serum-contributed growth factors from the gene expression patterns observed. The temperature switching is unique to the YAMC cells and their derivatives (mp53/Ras cells) used in our exemplar experiments. We measured gene expression of genes downstream of the perturbed gene in the absence of serum to be consistent with our prior work using YAMC and mp53/Ras cells (McMurray et al., 2008). For any other cell types or cell lines, it should be determined whether growth in the presence or absence of serum is more appropriate, as serum can have substantial impact on observed gene expression patterns. Following serum starvation, cells are harvested for RNA isolation and reverse transcription to generate cDNA that is used for TaqMan quantitative PCR assays.

12. Trypsinize and count perturbed cell populations to be used experimentally. Include an empty vector control in each experiment performed. For mp53/Ras cells, plate 250,000 cells per 10-cm dish.
13. Plate cells onto fresh collagen-coated dishes with 39°C media, which excludes interferon gamma and any eukaryotic-selective agents from the media (i.e., should be free of puromycin or similar). The media should contain anti-microbials, here kanamycin and gentamycin.
14. Allow cells to adhere and grow in 39°C CO_2_ incubator for 48 h.
15. Serum-starve cells by aspirating media from each dish and replacing with fresh 39°C media without serum.
16. Allow cells to grow for an additional 24 h in 39°C CO_2_ incubator.
17. Harvest cells following trypsinization. Inhibit trypsin activity using 39°C media with serum, then immediately collect each cell population into 15 mL conical tubes (one per perturbation) and pellet cells by centrifugation at 500 g for 5 min at 4°C.
18. Aspirate media and trypsin from cell pellets. Re-suspend cells in 5 mL 1× PBS (can be room temperature or ice-cold), then pellet again by centrifugation.
19. Aspirate PBS from cell pellets. Use immediately for RNA extraction OR snap freeze and store at −20°C.
  a. Cell pellets can be stored at −20°C for up to one month prior to RNA extraction.
20. Isolate RNA by following manufacturer’s protocol for the QIAGEN RNeasy Mini kit with On-Column DNase Digestion.
  a. Isolated RNA can be stored at −20°C for two years or longer if handled in an RNase-free manner and not exposed to repeated freeze-thaw cycles.
21. Prepare reverse transcription reactions:
  a. Denature 10 μg of each RNA sample to be used by incubation on a heat block or in a water bath at 70°C for 10 min.
  b. Plunge samples into ice to stop denaturation. Then add the components listed in the table below to each, keeping samples on ice.
    i. For multiple reverse transcription reactions, all components except for RNA and water can be made into a master mix for the appropriate number of reactions and aliquoted into RNA + water for necessary final concentration.
22. Once all components have been added, incubate samples at 42°C for 60 min.
23. Heat inactivate RT enzyme by shifting samples to 70°C for 10 min.
24. Hold samples on ice and proceed to PCR setup **OR** store at −20°C for long-term storage.

**Table:** Reverse transcription reaction (single reaction)

Reagent	Final concentration	Amount
Denatured RNA template	10 μg	Varies per sample
SuperScript II RT Buffer (5×)	1×	20 μL
DTT (100 mM)	10 mM	10 μL
dNTP mixture (10 mM each)	Dilute stock to 400 μM (100 μM each dNTP)	4 μL
Random hexamer primer (300 ng/μL)	300 ng	1 μL
RNaseOUT RNase Inhibitor (40 U/μL)	2 U/μL	5 μL
SuperScript II reverse transcriptase (200 U/μL)	4 U/μL	2 μL
ddH_2_O	Bring volume to 100 μL

**Table:** Reverse transcription reaction steps

Step	Temperature	Time
Denaturation (step 21a, above)	70°C	10 min
Plunge in ice (step 21b)	4°C	As necessary to prep master mix
Add master mix to each sample (step 21b)	4°C	As necessary
Incubate (step 22)	42°C	60 min
Heat inactivate RT enzyme (step 23)	70°C	10 min

Hold samples on ice and proceed to PCR setup OR store at −20°C for long term storage (step 24).

25. Prepare TaqMan qPCR assays reaction mix.
  a. The reactions were run on TaqMan Low Density Arrays, 384 well cards with primer pairs and probes pre-loaded into each well.
  b. Each reaction contained forward and reverse primer at a final concentration of 900 nM each and a TaqMan MGB probe (6-FAM) at 250 nM final concentration.
  c. For each sample:

**Table:** PCR reaction master mix

Reagent	Amount
cDNA template	82 μL
TaqMan Universal PCR Master Mix No AmpErase UNG (2×)	410 μL
Nuclease free H_2_O	328 μL

26. Load mixture into each of 8 ports on the array at 100 μL per port.
  a. Each individual sample of cDNA sample was processed on a separate card.

***Note:*** As described below in step 32 and further sections on TopNet modeling, four biological replicates of each perturbation were measured.

***Note:*** As noted in step 13 of the before you begin section, we considered a replicate to be an independently derived population of perturbed cells. Each replicate of a given perturbation had an empty vector control population of cells that was derived in parallel with the perturbed cell populations.

27. Seal arrays with a TaqMan Low-Density Array Sealer (Applied Biosystems) to prevent cross-contamination.
28. Run real-time amplifications on an ABI Prism 7900HT Sequence Detection System (Applied Biosystems) with a TaqMan Low Density Array Upgrade.

**Table:** PCR cycling conditions

Steps	Temperature	Time	Cycles
Sample loading	50°C	2 min	1
Initial denaturation	94.5°C	10 min	1
Denaturation	97°C	30 s	40 cycles
Annealing and extension	59.7°C	1 min	
Hold	4°C	forever

29. After real-time amplification is complete, obtain threshold cycle (C_t_) values via Sequence Detection Software (SDS, Applied Biosystems) following manufacturer’s instructions.
  a. SDS is graphical user interface software designed for use by wet-lab biologists. For this protocol, each sample was analyzed individually using this software package.
30. Export Ct values into a .csv file for use in the following steps.
  a. The Ct values from each sample were merged into a single table with probe sets as rows and perturbed samples or matched vector controls in each column.

### Prepare gene expression data for network modeling

**Timing: ∼10 min**

This step prepares the data for input to the network estimation algorithm.

31. Read in gene expression measurements for each perturbation and control experiment.
  a. As an example, here we read in Ct values from the crgnet experimental data R package available via github.

if(!require(remotes)){

 install.packages("remotes")

}

remotes::install_github('mccallm/crgnet')

library('crgnet')

data("crgdata")

### Normalization and missing data imputation

**Timing: ∼1 h**

**Figure 1:**
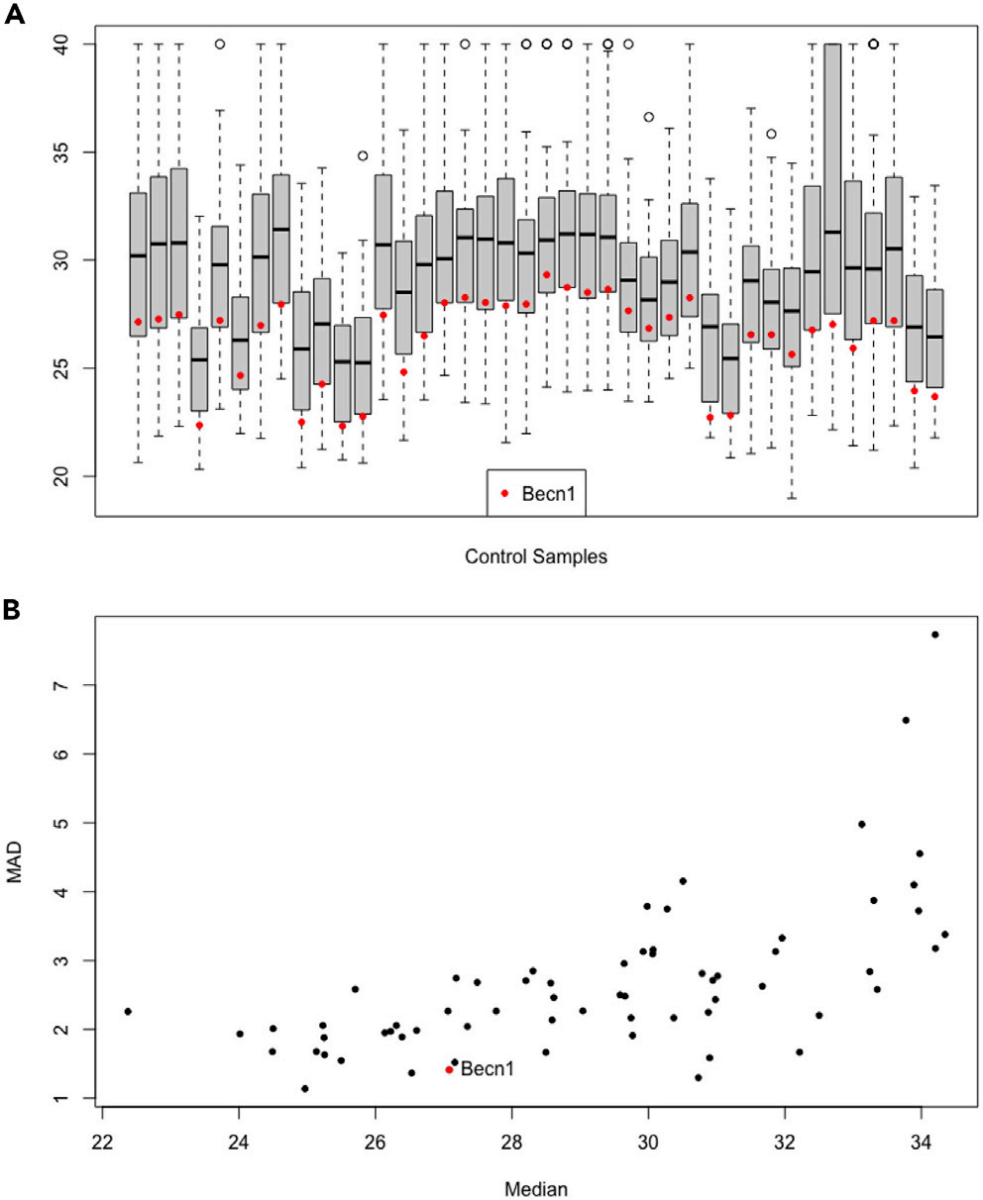
Rationale for the use of Becn1 to normalize qPCR data (A) shows the cycle threshold value (Ct) distribution for all measured genes in each control sample. Each box represents an independently derived cell population transduced with an empty vector. (B) shows the median average deviation (MAD) versus the median Ct value for each measured gene across all control samples. The red dot denotes Becn1 in both Panel A & B.

32. Select one or more control features to normalize the data. In this example, we use *Becn1* as a control because it appears to track the lower quantile of the distribution of expression across the controls reasonably well (Figure 1A) and has relatively low variability across the control samples compared to the other genes (Figure 1B). This suggests that normalizing to this house-keeping gene may perform well in these data.
  a. Examine variability in the distribution of expression across control samples. While some variability might be ascribed to the different control vectors, it is unlikely that the effect would be of this magnitude and consistency. Moreover, note that while there is substantial variability in the location of the expression distribution across the empty vector control samples, the range (e.g., IQR) remains relatively constant (except for one sample with the lowest overall expression that also had noticeably higher variability).
    ictl <- which(is.na(crgdata$pGene1))
    controlSamples <- assay(crgdata)[ ,ictl]
    boxplot(controlSamples, xaxt="n", xlab="Control Samples")
    becn1 <- controlSamples[rownames(controlSamples)=="Becn1",]
    points(becn1, pch=20, col="red")
    legend("bottom", pch=20, col="red", "Becn1")
    The house-keeping gene, Becn1, appears to track the lower quantile of the distribution of expression across the controls reasonably well (Figure 1A). This suggests that normalizing to this house-keeping gene may perform well in these data.
  b. Further examine the suitability of Becn1 for normalization by looking at the median absolute deviation (MAD) vs median expression.
    controlMedians <- apply(controlSamples, 1, median)
    controlMADs <- apply(controlSamples, 1, mad)
    plot(x=controlMedians, y=controlMADs, pch=20, ylab="MAD", xlab="Median")
    ind <- which(rownames(controlSamples)=="Becn1")
    points(x=controlMedians[ind], y=controlMADs[ind], pch=20, col="red")
    text(x=controlMedians[ind], y=controlMADs[ind], "Becn1", pos=4)points(becn1, pch=20, col="red")
    legend("bottom", pch=20, col="red", "Becn1")
    Becn1 has relatively low variability across the control samples compared to the other genes (Figure 1B).

33. Perform normalization and missing data imputation.
  a. Examine the relationship between the proportion of non-detects (those reactions failing to produce fluorescence values above a certain threshold) and the average observed expression value in the empty vector control samples. When the **plot** argument is set to **TRUE**, the **model_prep** function returns a scatterplot of the proportion of non-detects versus average expression for each gene across all control samples.
    crgprepL1 <- model_prep(crgdata, plot=TRUE)
    ***Note:*** The proportion of non-detects general increases for larger Ct values (lower gene expression). However, if this is not the case, an alternative imputation procedure, such as mean imputation, should be used. Additionally, if there are a large number of non-detects spread randomly across all measured genes, this may indicate poor sample quality.
    Additionally, the **model_prep** function converts the data to the qPCRset object format needed to impute the non-detects and returns normalization factors. Specifically, the **sampleType** field of the sample annotation contains a unique description of each experiment: which gene(s) were perturbed and the direction of perturbation. This is used to define replicates for use in the imputation.
  b. Treat non-detects as non-random missing data and impute using the R/Bioconductor package **nondetects** (McCall et al., 2014). This replaces missing values (non-detects) with an imputed values based on an estimated missing data mechanism as well as the expression values seen in replicate experiments.
    ***Note:*** Attempting to measure lowly expressed genes will result in a higher number of non-detects. As the number of non-detects increases, the imputation procedure becomes less reliable. Therefore, while there isn’t a universal threshold for the applicability of the non-detects imputation, there is a trade-off between the ability to measure lowly expressed genes and the reliability of the imputation. Often this can be addressed by increasing the number of replicates such that the number of observed values for each gene is sufficiently large.
    crgdataImputed <- qpcrImpute(crgprepL1$object, groupVars="sampleType")
    ***Note:*** This function takes a while to run.
  c. Normalize the data. Here, we normalize the data to the house-keeping gene, Becn1.
    library(HTqPCR)
    crgdataNorm <- normalizeCtData(crgdataImputed, deltaCt.genes="Becn1")
    exprs(crgdataNorm) <- -exprs(crgdataNorm)
    If we were normalizing to multiple control genes, we would first calculate the mean expression of the control genes then proceed as above.
    ***Note:*** After normalization, we have lost the cycle threshold (Ct) interpretation but retained the inverse relationship between expression values (on the Ct scale) and the amount of transcript in the sample. Therefore, we consider the negative ΔCt value as our measure of normalized expression.

### Quantify response to perturbations

**Timing: ∼30 min**

In the previous sections we have dealt with non-detects (missing values) and normalized the data. We now turn out attention to assessing which genes are up- or down-regulated in response to each perturbation.

**Figure 2:**
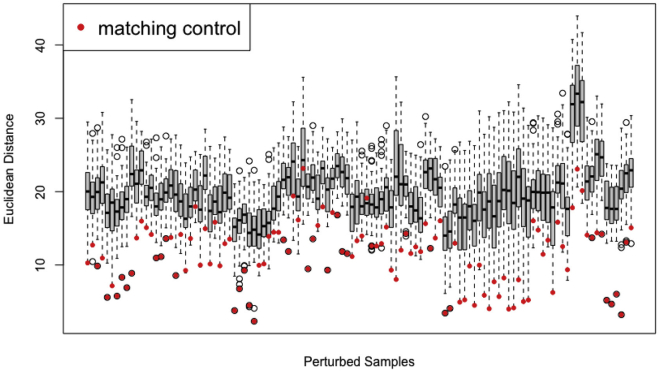
Matched control samples capture potential batch effects For each perturbed sample (x-axis), the distribution of Euclidean distances from that the vector of Ct values for that perturbed sample to each control sample is shown. The matched control sample for each perturbed sample is highlighted in red. In general, a perturbed sample is most similar to its corresponding control sample, suggesting that matched control and perturbed samples are similarly affected by potential batch effects.
  c. Calculate paired ΔΔCt values by computing the difference in expression between each perturbed sample and its corresponding control sample from the same batch by running calculate_ddCt().
    ddCt <- calculate_ddCt(crgdataNorm)
  d. One final check of the non-detect imputation procedure from before is to examine the distribution of residuals stratified by the presence of imputed non-detect values. These can exist in either the perturbed sample, the control sample, or both samples.
    resids <- examine_residuals(ddCt, plot=TRUE)
    Here, we see what one would expect: a median of zero and roughly equal spread when there are no non-detects, a median slightly below zero when there is a non-detect in only the perturbed sample, and a median slightly above zero when there is a non-detect in only the control sample (Figure 3).

34. Calculate ΔΔCt values that quantify the change in expression from controls for each perturbation.
  a. Examine whether a given perturbed sample is more similar to the control sample from the same batch then to control samples from other batches by running the **check_matched_controls** function. If there is a batch-effect, it is advantageous to compare each perturbed sample to the control sample from the same batch.
    check_matched_controls(crgdataNorm)
  b. In almost all cases, the control sample from the same batch is the most similar to the perturbed sample (Figure 2). This suggests that there is a difference between batches and motivates the calculation of paired ΔΔCt values as our measure of normalized change in expression in response to each perturbation. If there were no difference between batches, the distance from a given perturbed sample to its matched control (points in Figure 2) would be randomly distributed within the set of pairwise differences. An eyeball test is often sufficient to assess this; however, a permutation-based statistical test could also be used. If a batch effect does not exist, then we can simply compare each perturbed sample to the average of all the control samples to compute unpaired ΔΔCt values.

**Figure 3:**
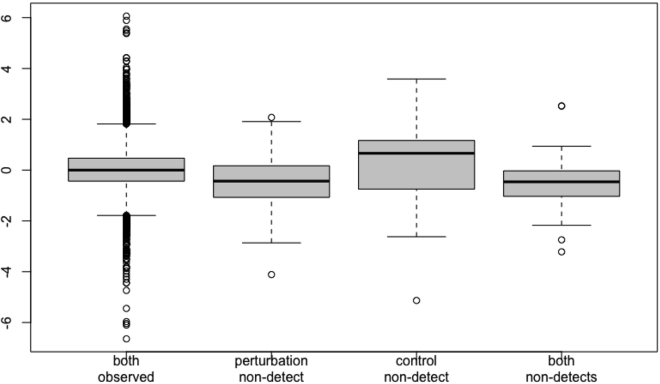
The effect of non-detects on estimates of gene expression The distribution of residuals stratified by the presence of imputed missing values (non-detects) is shown. Note that on average, a non-detect in the perturbed sample results in slightly negative residuals, while a non-detect in the control sample results in slightly positive residuals.
  e. Calculate approximate z-scores for each perturbation after removing any control genes. Here, we remove the control gene Becn1.
    ddCt_no_ctl <- ddCt[-which(rownames(ddCt)=="Becn1"), ]
    zscores <- calculate_zscores(ddCt_no_ctl)
  f. Calculate the probability of up- / down-regulation in response to each perturbation by fitting a uniform / normal / uniform mixture model.
    probabilities <- calculate_probs(zscores)
  g. Filter genes that were measured but are not perturbed in any experiment. While these genes are useful in modeling the missing data mechanism for non-detect imputation and estimating probabilities of up- / down-regulation, they will not be used in the subsequent network modeling and can be removed at this point. Here, we also filter several samples that do not represent the type of perturbation being modeled, a single perturbation back to normal expression levels.
    perts <- gsub(":.+","",colnames(probabilities))
    ind <- which(!rownames(probabilities) %in% perts)
    probabilities <- probabilities[-ind, -c(1,9,12,13,20,23)]
    -c(1,9,12,13,20,23)
  h. Format the probabilities for input to the network model fitting algorithm.
    networkInputData <- format_network_input(probabilities)
    ***Optional:* Connectivity graph analysis**. Either the z-scores or probabilities could be thresholded to produce a connectivity graph, in which nodes represent genes and directed edges denote that perturbation of the parent gene results in a change in expression of the child gene. Here, we create a connectivity graph based on the probabilities by thresholding the probabilities at an absolute value of 0.5. In other words, if the probability that perturbation of gene A results in a change in expression of gene B exceeds 0.5 then an edge from gene A to gene B is included in the connectivity graph.
    probs <- networkInputData$ssObj
    colnames(probs) <- rownames(probs)
    probs[which(abs(probs)<0.5, arr.ind=TRUE)] <- 0
    diag(probs) <- 0
    Next, we use the **network** package to create and plot the connectivity graph (Figure 4). We also add data on tumor inhibition and direction of response.
    library(network)
    cgraph <- network(t(probs), matrix.type="adjacency",
         ignore.eval=FALSE, names.eval="probs")
    data("tumor_inhibition")
    set.vertex.attribute(cgraph, "tumor_inhibition", tumor_inhibition$TumorEffect)
    set.edge.attribute(cgraph, "direction", sign(get.edge.attribute(cgraph,"probs")))
    plot(cgraph, displaylabels=TRUE, mode="circle", boxed.labels=TRUE,
     label.bg=ifelse(get.vertex.attribute(cgraph, "tumor_inhibition")=="Smaller", "yellow", "grey"),
     edge.col=ifelse(get.edge.attribute(cgraph, "direction")==1, "red", "blue"))
    legend("topleft", c("Tumor Inhibitory",
     "Not Tumor Inhibitory"), title="Node Color",
     fill=c("yellow","grey"))
    legend("topright", c("Up-regulation", "Down-regulation"), title="Edge Color",
     col=c("red","blue"), lty=1, lwd=5)

**Figure 4:**
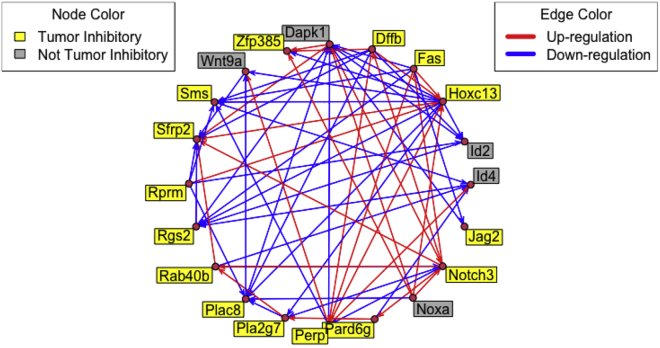
A connectivity graph showing the effect of perturbation for 20 CRGs Arrows originating from a node denote the effect of perturbation of that node. Arrows terminating at a node represent the effect on that node, and the edge color denotes up-regulation (red) or down regulation (blue). The tumor inhibitory effect of each perturbation is encoded by the color of each node, tumor inhibitory (yellow) or not tumor inhibitory (grey).

### Perform network modeling

**Timing: ∼5 days**

This step estimates the network of interactions.

35. Network modeling. Use a ternary network model that accounts for the dynamic nature of gene regulatory networks and facilitates the evaluation of uncertainty to model a gene regulatory network. Specifically, use a parallel tempering algorithm (Swendsen and Wang, 1986) to search the model space for networks that produce attractors that are most similar to the observed steady state data. Pseudocode for the network modeling algorithm is supplied in Methods S1, while a reproducible workflow of the analyses in this paper is included as the vignette of the crgnet package.
  ***Note:*** Unlike previous approaches, here we have incorporated uncertainty in the differential expression estimates via probabilities of up- / down-regulation. This allows the network model to give more weight to data points with higher certainty. Additionally, the ability to produce non-integer network scores eased transitions between network models and significantly decreased computational time.
  a. Here, we show example code to fit a network using 1,000,000,000 cycles in parallel across 20 processors with temperatures ranging from 0.001 to 1. These parameters should be chosen such that either the network reaches a score of zero (indicating a fit that perfectly explains the observed data) or until additional cycles do not produce a reduction in the network score. The code used to produce these network fits is shown below:
    library("ternarynet")
    data("crgnet_scores")
    results <- parallelFit(experiment_set=crgnet_scores,
        max_parents=4,
        n_cycles=1e9,
        n_write=10,
        T_lo=0.001,
        T_hi=1,
        target_score=0,
        n_proc=20,
        logfile="tnet-fit.log"),
        seed=as.integer(112358)
    )
    ***Note:*** The computational time required to generate these network models is substantial: each independent network fit presented in McMurray et al. (2021) took approximately 12 h to run in parallel on 20 compute nodes. However, less than 1 GB of RAM was sufficient to fit these network models.

### Create network summaries and visualization

**Timing: ∼1 h (∼1 month with optional steps included)**

This step prepares the data for input to the network estimation algorithm.

36. Summary statistics can be computed by calculating the proportion of networks in which a given feature or features are present. One can also examine the transition functions, attractors, and trajectories all stored in the **fits** object. One of the most common ways to visualize a network model is to present the topology. Here we calculate proportion of networks in which a given gene is a parent of another given gene.

data(networkInputData)

data(networkFits)

topo <- topology(fits)

rownames(topo) <- rownames(networkInputData$ssObj)

colnames(topo) <- rownames(networkInputData$ssObj)

This information can be exported to Cytoscape or other network visualization software to create a graphical representation of these results.

***Optional:*** One question of interest is whether it is significant that one can obtain a low scoring network model. This can be examined by permuting the network input data. For each gene, permute its response to all of the experiments while retaining the number of experiments to which each gene responds. In other words, the number of parents in a connectivity graph remains constant but which other genes are parents’ changes. Then fit a network model to the permuted data using the same parameters as above and repeat this process to generate multiple permuted fits. Here, we load precomputed permuted fits from the crgnet package.

data(networkFits)

data(permutedNetworkFits)

real_scores <- sapply(fits, function(x) x$unnormalized_score)

permuted_scores <- sapply(pfits, function(x) x$unnormalized_score)

hist(permuted_scores, breaks=25, main="",

xlab="Network Scores")

rug(real_scores, lwd=3)

**Figure 5:**
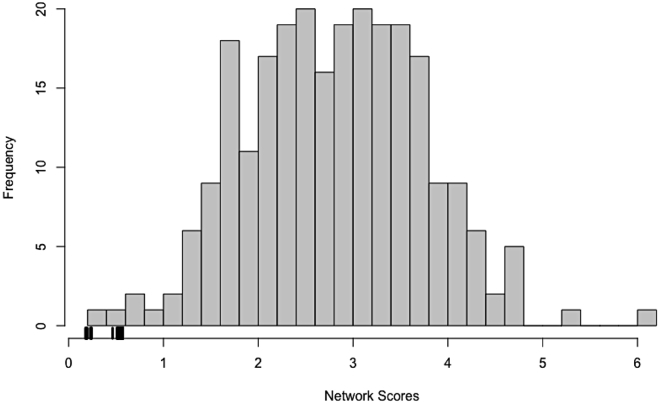
Network scores produced by real data are lower than those produced by permuted data For each gene, its response to all of the experiments was permuted while retaining the number of experiments to which each gene responds. In other words, the number of parents in a connectivity graph was held constant but the identities of the parent genes were changed. A network model was then fit to the permuted data using the same parameters as used for the real data, and this process was repeated 100 times to generate multiple permuted fits. A histogram of network scores corresponding to the permuted fits is shown. Tick marks on the x-axis denote the scores produced by the real data network fits.

The network scores based on the real data are less than nearly all the scores based on the permuted data (Figure 5; empirical p-value ≤ 0.01). With the current network constraints, we can obtain good scores for the real data but not for the permuted data. This suggests that we are not extensively overfitting these data and that the current network constraints are reasonable.

***Optional:*** To examine whether we could obtain similar fits with a simpler network model, one can vary the in-degree and compare model fits. As an example, we reran the network modeling algorithm with the max_parents reduced from 4 to 3 and also considered a more complex model by increasing the max_parents parameter to 5. We ran both models on permuted data as well as the real data.

data(networkFits)

data(networkFitsIndeg3)

data(networkFitsIndeg5)

indeg4_scores <- sapply(fits, function(x) x$unnormalized_score)

indeg3_scores <- sapply(fits3, function(x) x$unnormalized_score)

indeg5_scores <- sapply(fits5, function(x) x$unnormalized_score)

data(permutedNetworkFits)

data(permutedNetworkFitsIndeg3)

data(permutedNetworkFitsIndeg5)

indeg4_permuted_scores <- sapply(pfits, function(x) x$unnormalized_score)

indeg3_permuted_scores <- sapply(pfits3, function(x) x$unnormalized_score)

indeg5_permuted_scores <- sapply(pfits5, function(x) x$unnormalized_score)

plot(x=jitter(rep(c(1:6), c(length(indeg3_scores), length(indeg3_permuted_scores), length(indeg4_scores), length(indeg4_permuted_scores), length(indeg5_scores), length(indeg5_permuted_scores)))),

 y=c(indeg3_scores, indeg3_permuted_scores, indeg4_scores, indeg4_permuted_scores, indeg5_scores, indeg5_permuted_scores),

 ylab="Network Score", xlab="", xaxt="n")

axis(1, line=1.5, at=c(1.5,3.5,5.5), labels = c("In-degree 3", "In-degree 4", "In-degree 5"), tick=FALSE, cex.axis=1.25)

axis(1, at=c(1:6), labels = rep(c("Real", "Permuted"), 3))

**Figure 6:**
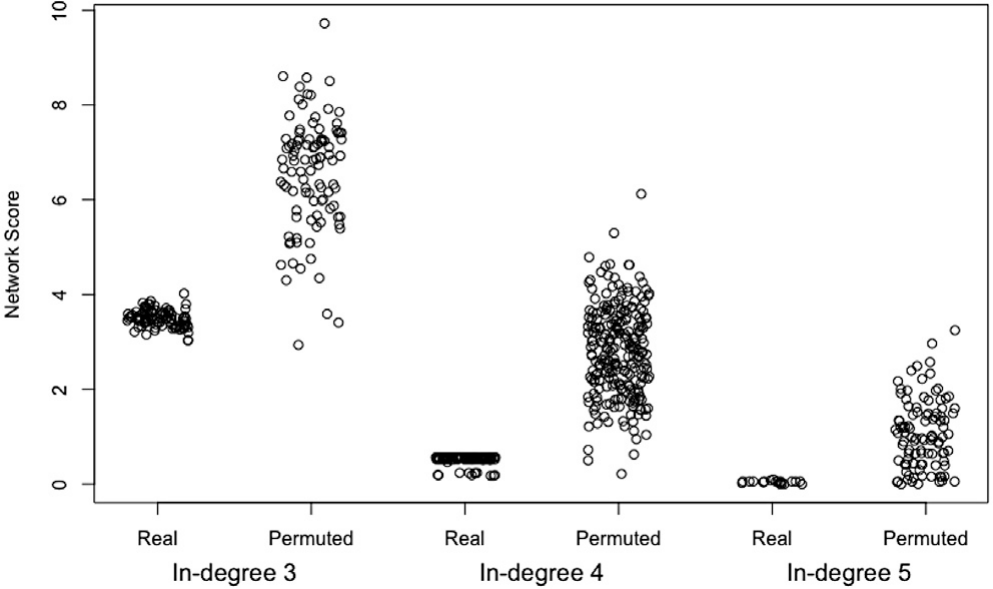
The effect of different in-degree limits on network scores and the ability to distinguish between real and permuted data The network modeling algorithm was run with in-degree limits of 3, 4, and 5 on both real and permuted data. Larger in-degree limits produce generally better scores, and the real data produce better scores than the permuted data across all three in-degree limits.

***Note:***Figure 6 illustrates that increasing the in-degree cap from 3 to 4 results in a sizeable reduction in the model score; however, increasing the in-degree cap to 5 produces only a modest improvement (the in-degree 4 network models already do quite well). Regardless of the in-degree cap, better scores were achieved using the real data (as expected). The separation between real and permuted scores is greatest for an in-degree cap of 4. This lends further support to the choice of a maximum in-degree of four for these data.

***Optional:*** Comparison between network models generated using different in-degree thresholds. Here, we demonstrate the effect of the maximum in-degree on the resulting network topology. Note that a larger in-degree threshold will produce a network with more edges. Of primary interest is whether the high confidence edges are retained for varying in-degrees.

data(networkFits)

data(networkFitsIndeg3)

data(networkFitsIndeg5)

topo_i4 <- topology(fits)

topo_i3 <- topology(fits3)

topo_i5 <- topology(fits5)

rownames(topo_i3) <- colnames(topo_i3) <-

 rownames(topo_i4) <- colnames(topo_i4) <-

 rownames(topo_i5) <- colnames(topo_i5) <- rownames(networkInputData$ssObj)

par(mfrow=c(1,2))

plot(x=topo_i4, y=topo_i3, pch=20,

 xlab="In-degree 4 edge proportions",

 ylab="In-degree 3 edge proportions",

 main=paste0("Correlation = ", round(cor(as.vector(topo_i3), as.vector(topo_i4)), digits = 2)))

abline(v=0.8)

plot(x=topo_i4, y=topo_i5, pch=20,

 xlab="In-degree 4 edge proportions",

 ylab="In-degree 5 edge proportions",

 main=paste0("Correlation = ", round(cor(as.vector(topo_i4), as.vector(topo_i5)), digits = 2)))

abline(v=0.8)

### Testing tumor formation capacity of cells with two genetic perturbations based on features of the network model

**Timing: 5–6 weeks**

Here, we describe the process of measuring tumor formation in perturbed mp53/Ras cell populations and linking these measurements to features of the network model. This is done by perturbation of multiple target genes selected from among the network nodes in mp53/Ras cells. Subsequently, the perturbed cell populations are implanted into allogeneic, immune compromised mice and tumor growth is measured by monitoring tumor size over time. We perform these studies in CD-1 nude mice (Crl:CD-1-Foxn1nu, Charles River Laboratories, purchased at 6–8 weeks of age), but a number of other immune compromised mouse strains could be used (e.g., NOD/SCID animals).

37. Follow steps 1 through 16 in the step-by-step method details section to generate cell populations with desired perturbation of combinations of target genes selected based on network modeling results.
38. Trypsinize cells following 48 h of growth at 39°C. Pellet cells, wash with 1× PBS (as in step 17 above) BUT DO NOT FREEZE.
39. Re-suspend cell pellets in 500 μL of RPMI with no additives and keep on ice. Count cells with an automated cell counter or hemacytometer.
40. Dilute cells to 5 × 10^5^ per 100 μL in RPMI 1640 media with no additives.
41. Implant 100 μL of re-suspended cells into each flank of each experimental animal (i.e., two implantations per animal).
  a. Animals should be implanted with only one type of cell population – vector control or a single gene perturbation per animal.
    i. For experiments in McMurray et al. (2021), six implantations were done using cells from each perturbed cell population. In parallel, six implantations were done with cells from a freshly derived empty vector control population included in each experiment.

***Note:*** At implantation, mark or number animals in some manner (ear punch, tattoo or alternative method) to keep track of tumor growth per animal per week.

42. At 7, 14, 21 and 28 days post-implantation, measure tumor diameter by caliper at two distinct points of each tumor for each animal on both flanks independently.
43. Use tumor diameter data to calculate tumor volume using the standard formula for volume of a sphere (volume=(4/3)πr^3^).
44. Examine and quantify the association between predicted gene interactions and phenotypic outcomes (here tumor size).

## Expected outcomes

TopNet provides an integrated pipeline to process data from perturbation experiments, generate network models consistent with the observed data, and summarize and visualize these networks. The outputs of this pipeline are the network topology and set of transition functions that define the network, as well as several parameters that can be used to assess the quality of the network model. The output of TopNet can also be readily visualized in Cytoscape and other network visualization tools. As demonstrated in (McMurray et al., 2021), summaries of the network topology can be analyzed in concert with phenotypic variables of interest (e.g., tumor growth) to pinpoint biologically relevant aspects of the network architecture.

## Limitations

### Generating cell populations with gene perturbations via retroviral vectors

The pBabe retroviral packaging system cannot effectively transduce gene expression inserts larger than about 7,000 bp, due to the retroviral capsid particle size and thus the amount of nucleic acid that can be packaged.

The pSuper-retro vector system delivers shRNA-expressing constructs to the target cell population. Despite following specific shRNA design guidelines, not every shRNA insert will effectively reduce the expression of the targeted gene. Thus, it is necessary to test multiple shRNAs that target distinct sequences in a given gene to identify those that successfully knock down the gene’s expression.

shRNA is known to have potential off-target effects. Thus, it is advisable to independently derive cell populations using constructs that target distinct sequences within a given gene of interest to ensure that observed phenotypes are consistent between constructs and target sequences, and thus unlikely to result from off-target effects of a single vector.

### Control of perturbagen expression

When perturbing gene expression using these systems, there is little control over the amount of perturbation that is achieved. For experiments described in McMurray et al. (2021), every derivation of each perturbed mp53/Ras cell population was compared to empty vector-infected mp53/Ras control populations derived in parallel. Expression of the perturbed gene was also compared to parental YAMC expression levels to discern whether the gene’s expression was re-set in perturbed mp53/Ras cells to YAMC levels, our target for these experiments. As described previously (McMurray et al., 2008, 2021), cell populations that did not meet this standard were either excluded from further studies or results were interpreted in the context of known over-expression.

### Flexibility of TopNet modeling

TopNet requires data in which each node in the network is both measurable and perturbable. In McMurray et al. (2021), the nodes of the network were genes measured via qPCR and perturbed using lentiviral vectors. While applicable to more than just gene expression data, TopNet cannot currently handle data in which the perturbations are not also the nodes of the network, e.g., chemical compound perturbation.

### Scalability of TopNet modeling

The size of the network model space scales rapidly with both the number of nodes and the maximum number of parents per node (the in-degree limit). This means with current computational resources, networks larger than 1,000 nodes are likely infeasible to estimate. However, advances in computational power and improvements in search algorithms will likely reduce this limitation over time.

## Troubleshooting

### Problem 1

Inability to derive a polyclonal cell population following retroviral infection and drug selection.

This problem can arise for several reasons that all lead to the same problem – insufficient expression of the resistance gene encoded by the vector, such that transduced cells succumb to drug selection. This can happen due to 1) poor transduction of cells with the retroviral supernatants, 2) insufficient viral titers to generate an efficient infection, 3) sensitivity of the particular cell line / cell type to the drug of choice or 4) failing to wait for cells to undergo at least one cell division before beginning drug selection (retroviruses require cell division for integration into the host cell genome).

### Potential solution

The solution depends on the source of the problem.

• Poor transduction (1) may be improved by increasing the amount of polybrene mixed into the retroviral supernatants during infection. This is limited by overt toxicity of this additive, so test a range of dosages on the cells of interest to identify a maximum concentration.
• Insufficient viral titers may be overcome by improving transfection efficiency to produce more retrovirus and/or by using more supernatant or concentrating supernatants to provide more infectious units to the target cells.
• Cell sensitivity to the selection agent can be examined by testing a range of drug dosages on uninfected cells. Choose the minimum dose to achieve the desired effect – either cytotoxicity or cytostatic effects.
  ○ Alternatively, inserts can be cloned into vectors with different drug selectable markers to allow for use of a selection agent with less baseline toxicity to the target cells.
• If it is necessary to move rapidly from infection to selection, use of lentiviral vectors may be preferable. Lentiviruses are able to infect non-dividing cells and thus generally have higher infection efficiencies than retroviral vector systems.

### Problem 2

The network returns a null model after a short runtime.

This could arise in two situations: (1) the experimental data itself does not contain any connections, or (2) the input data to the network fitting algorithm was incorrectly formatted.

### Potential solution

First, examine a connectivity graph to assess whether any nodes in the network response to perturbation of other nodes. If the connectivity graph contains no edges, then it is possible that the perturbations that were performed did not produce changes in any of the measured nodes. However, if the connectivity graph contains edges, the input data to the network fitting algorithm was likely incorrectly formatted. An example of correctly formatted data is available in the **crgnet** package and helper functions to produce correctly formatted data are available in the **ternarynet** package.

### Problem 3

The network model never runs to completion.

This problem may arise when the number of nodes in the network or the number of possible parents for each node (the in-degree limit) are too large for the available computation resources used to run the network fitting algorithm.

### Potential solution

**Figure 7:**
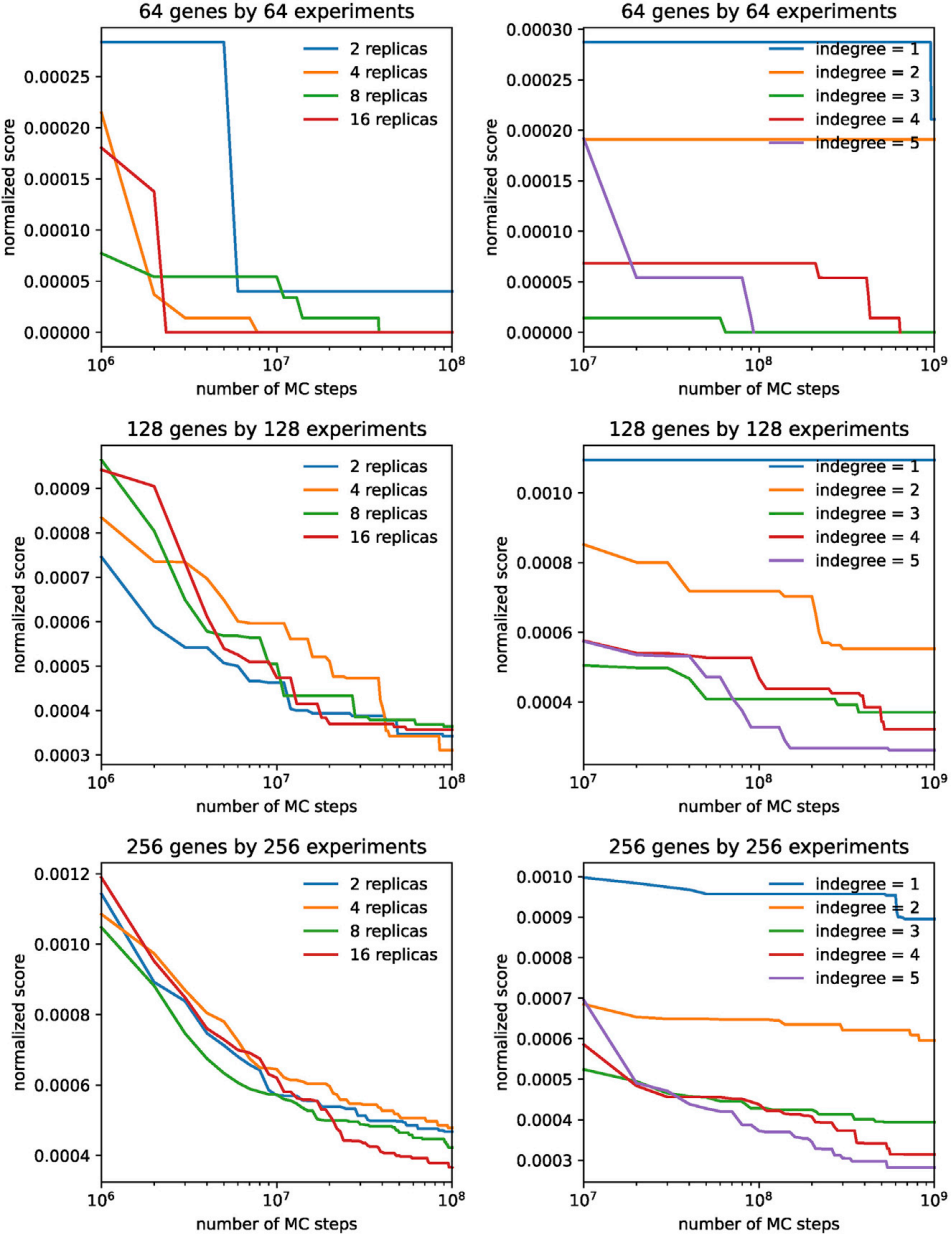
The effect of number of replicas and in-degree limit on network modeling The normalized score (where the normalization factor is the product of the number of genes and experiments) vs the number of Monte Carlo steps for networks of size 64, 128, and 256 genes and perturbations. Left: varying the number of replicas, keeping indegree fixed at 4. Right: varying the indegree from 1 to 5, keeping the number of replicas fixed at 3.

This problem can be solved by: (1) reducing the size of the network model (either the number of nodes or the in-degree limit) or (2) increasing the available computational resources, e.g., running the code on a high performance computing cluster or in the cloud. Additionally, if running the network fitting algorithm using the serial simulated annealing search algorithm, it could help to instead use the parallel replica exchange Monte Carlo search algorithm. Figure 7 shows the normalized network scores (zero corresponds to a perfect fit) for varying network sizes (64, 128, and 256 nodes), vary in-degree limits of 1–5, and varying number of replicas using in the replica exchange Monte Carlo search algorithm.

### Problem 4

The network model returns a score far from zero.

This problem may arise for several reasons: (1) the input scores are on a different scale, (2) the network search algorithm was not allowed to run for a sufficient length of time, (3) the data contain seemingly contradictory responses to the perturbation experiments.

### Potential solution

This will likely take some investigation. First, to check if possibility (1) is correct, one can calculate the minimum possible score from the input data. If this is far from zero, an error may have occurred in generating the scores. Alternatively, if using a custom scoring function that simply produces scores on a different scale, one could consider shifting or rescaling the input scores. Second, to check if possibility (2) is the issue, simply run the network model for more cycles and see if the scores improve. One can examine the scores as a function of cycles (see Figure 7 for examples) to determine whether the scores are still decreasing when the maximum number of cycles is reached. Finally, possibility (3) is the most challenging to assess but is a likely cause if possibilities (1) & (2) are ruled out. In this situation, the best approach is to increase the in-degree limit of the network model.

### Problem 5

The network model returns few if any high confidence edges.

This problem may arise when there is insufficient information to resolve uncertainty in the network model space.

### Potential solution

The most direct solution would be to perform additional perturbation experiments to reduce modeling uncertainty. Alternatively, one could restrict the model space algorithmically by decreasing the maximum in-degree allowed.

## Resource availability

### Lead contact

Further information and requests for resources and reagents should be directed to and will be fulfilled by the lead contact, Matthew McCall (matthew_mccall@urmc.rochester.edu).

### Materials availability

Plasmids generated in this study are available upon request.

Primer sequences and TaqMan probe sets are tabulated in the key resources table.

There are restrictions to the availability of YAMC cells due to materials transfer agreement.

## Data Availability

All qPCR data are available in the supplemental information of McMurray et al., Cell Reports, Dec 2021. Tumor volume data reported in this paper will be shared by the lead contact upon request. All original code to reproduce all statistical analyses is available in the crgnet R package available at: https://github.com/mccallm/crgnet/ and the ternarynet R/Bioconductor package, available at: https://bioconductor.org/packages/ternarynet/. The pseudocode for the network modeling algorithm is supplied in Methods S1, while a reproducible workflow of the analyses in this paper is included as the vignette of the crgnet package. Any additional information required to reanalyze the data reported in this paper is available from the lead contact upon request.
