# Supplementary material for: Protocol to use TopNet for gene regulatory network modeling using gene expression data from perturbation experiments
Source: STAR Protoc. 2022 Sep 30;3(4):101737. doi: 10.1016/j.xpro.2022.101737 (PMC9529586; doi:10.1016/j.xpro.2022.101737)
Supplement: Document S1. Methods S1 [file mmc1.pdf]

Fitting ternery network models by replica exchange

The `retnfit` package contains a parallel implementation of the replica exchange Monte Carlo algorithm (Swendsen and Wang, 1986), also known as parallel tempering, for fitting ternary network models. Pseudocode describing the replica exchange algorithm is given below. Here,

- γ denotes a random deviate drawn from the uniform distribution on [0,1)
- ν, ν' denote nodes chosen at random
- $\pi(i)$ denotes one of the parents, chosen at random, of a node i
- $\alpha(i)$ denotes one of the outcomes, chosen at random, for the transition function of node i
- σ is a possible outcome (-1, 0, or 1) chosen at random
- the cost function (the deviation of a given network from target data) is the same as described in reference 2
- For each trial move, each initial condition is advanced according to the transition functions until either a repetition is detected, or a maximum number of states is reached, in which case a large value for the cost function is assigned so that the move will not be accepted.
- the numbers of parents and transition function outcomes changed per Monte Carlo cycle is adjusted dynamically based on the fraction of those moves that are accepted, to reach a target acceptance probability of 0.5. This adjustment is done every $N_{\mathrm{AdjustMoveInterval}}$ cycles, which is set to 7001
- exchanges between replicas are attempted every $N_{\mathrm{ExchangeInterval}}$ cycles, which is set to 1000
- the algorithm terminates if either a target score or the maximum number of cycles is reached.

$N_{\mathrm{ParentMoves}} := 1$
$N_{\mathrm{OutcomeMoves}} := 1$
for i := 1 **to** N_{replicas} **do in parallel**:
  set-network-for-replica-i-to-initial-state
  $T_i := T_{\mathrm{hi}} \left(\frac{T_{\mathrm{hi}}}{T_{\mathrm{lo}}}\right)^{(i-1)/(N_{\mathrm{replicas}}-1)}$
for j := 1 **to** N_{cycles} **do**:
  for i := 1 **to** N_{replicas} **do in parallel**:
    if j **mod** $2 = 1$:
      for k := 1 **to** $N_{\mathrm{ParentMoves}}$
        $\pi(\nu) := \nu'$
    else:
      for k := 1 **to** $N_{\mathrm{OutcomeMoves}}$

$\alpha(\nu) := \sigma$
$E_i^{(0)} = E_i$
E_i = 0
for k := 1 **to** $N_{\text{InitialStates}}$:
set-state-to-initial-state-k
for l := 1 **to** $N_{\text{MaxStates}}$
advance-state-k
if repetition-detected:
$E_i = E_i +$ difference-with-target-values
break
if $l = N_{\text{MaxStates}}$:
$E_i =$ large-value
break
if $\gamma < \exp\left(-\left[E_i - E^{(0)}\right]/T_i\right)$:
accept-move
else:
restore-original-network-for-replica-i
if j **mod** $N_{\text{ExchangeInterval}} = 0$:
for i := 1 **to** $N_{\text{replicas}} - 1$ **do in parallel**:
E_i := cost-function-for-replica-i
E_{i+1} := cost-function-for-replica-i+1
if $\gamma < \exp\left(-\left[E_{i+1} - E_i\right]\left[\frac{1}{T_i} - \frac{1}{T_{i+1}}\right]\right)$:
exchange-networks-for-replicas-i-and-i+1
if best-score-for-any-replica $\leq$ target-score:
break
if j **mod** $N_{\text{AdjustMoveInterval}} = 0$:
if fraction-of-parent-moves-accepted > 0.5:
$N_{\text{ParentMoves}} := N_{\text{ParentMoves}} + 1$
else if $N_{\text{ParentMoves}} > 1$:
$N_{\text{ParentMoves}} := N_{\text{ParentMoves}} - 1$
if fraction-of-outcome-moves-accepted > 0.5:
$N_{\text{OutcomeMoves}} := N_{\text{OutcomeMoves}} + 1$
else if $N_{\text{OutcomeMoves}} > 1$:
$N_{\text{OutcomeMoves}} := N_{\text{OutcomeMoves}} - 1$
